# Complete Genome Sequence of Salmonella Phage vB_SenA_SM5, Active against Multidrug-Resistant Salmonella enterica Serovar Typhi Isolates

**DOI:** 10.1128/mra.00309-22

**Published:** 2022-06-15

**Authors:** Naveen Chaudhary, Dharminder Singh, Ravi Kumar Maurya, Balvinder Mohan, Neelam Taneja

**Affiliations:** a Department of Medical Microbiology, Postgraduate Institute of Medical Education and Research, Chandigarh, India; DOE Joint Genome Institute

## Abstract

Phage vB_SenA_SM5, active against multiple isolates of multidrug-resistant Salmonella enterica serovar Typhi, was isolated from the sewage water of a tertiary-care referral hospital in Chandigarh, India. It has a 154.4-kb-long double-stranded DNA genome, belongs to the family *Ackermannviridae*, and is closest to Salmonella phage Chennai, which was isolated in southern India.

## ANNOUNCEMENT

Here, we present the complete genome sequence of phage vB_SenA_SM5, which exhibits lytic activity against multiple strains of Salmonella enterica serovar Typhi resistant to ampicillin, azithromycin, ciprofloxacin, tetracycline, and nalidixic acid (Table 1).

**TABLE 1:** Antibiotic sensitivity pattern of Salmonella enterica serovar Typhi isolates used to test the activity of phage vB_SenA_SM5^*a*^

Strain	Ampicillin	Chloramphenicol	Co-trimoxazole	Azithromycin	Ciprofloxacin	Tetracycline	Ceftriaxone	Nalidixic acid	Activity^*b*^
SM5	S	S	S	S	S	S	S	S	+
SM4	R	S	S	R	R	S	S	R	+
SM 11	S	S	S	R	S	R	S	S	+
SM8	S	S	S	R	R	R	S	R	+
SM1	S	S	S	S	R	S	S	R	+
SM3	S	S	S	I	S	R	S	I	+
SM9	S	S	S	R	S	S	S	R	+
SM6	R	S	S	S	R	S	S	R	+
SM2	S	S	S	S	S	S	S	R	−

a S, susceptible; R, resistant; I, intermediate.

b −, no activity or turbid spot; +, lytic or clear spot.

Three sewage samples were collected from the main sewage drain at the Postgraduate Institute of Medical Education and Research (PGIMER), Chandigarh, India (30.7333°N, 76.7794°E), a 2,200-bed tertiary-care hospital. The samples were incubated with a culture of S. Typhi strain SM5 in Trypticase soy broth (TSB) at 37°C for 20 h and centrifuged at a speed of 4,000 rpm for 15 min. The supernatants were filtered with 0.22-μm syringe filters, and the filtrates were used for a plaque assay (1). A single plaque was selected and propagated twice as described by Valente et al. (2). The phage preparation was concentrated by the addition of polyethylene glycol (10% PEG 8000), followed by ultracentrifugation at 55,000 rpm for 2 h (3). The final phage volume was resuspended in 1 mL of SM buffer (5.8 g/L NaCl, 100 mM MgSO_4_·7H_2_O). Phage DNA was extracted using a DNA isolation kit (Norgen Biotek, Canada); a genome sequencing library was prepared using a NEBNext Ultra kit, and sequencing was executed on an Illumina NovaSeq 6000 instrument (4). The DNA sequencing produced 10,467,332 paired-end (150-bp-long) raw reads for an average depth of 10,000×. Quality assessment of the raw Illumina paired-end reads was performed using FastQC v 0.11.9. *De novo* assembly of the generated reads was performed using the Iterative Virus Assembler (IVA) v1.0.8 (5). The Rapid Annotation Search Tool (RAST) was used to perform genome annotation (6). The predicted genes were compared with the UniProtKB/Swiss-Prot database using the BLASTX program with an E-value cutoff of 10^−3^. The genome completeness of the DNA contig obtained was determined using the tools CheckV and PhageTerm (7, 8). The genes encoding tRNAs were scanned using the tool tRNAscan-SE (9). All tools were run with default parameters. The CheckV and PhageTerm analysis showed that the resulting contig is complete and lacks cohesive genome ends. The phage genome is double stranded and linear and contains 157,408 bp, with a GC content of 44.4%. A BLASTn nonredundant (nr) database similarity analysis showed that the phage vB_SenA_SM5 genome is closest (query coverage, 97%; identity, 99.48%) to Salmonella phage Chennai (GenBank accession no. MN953776). A total of 211 open reading frames (ORFs) were identified; only 75 of the ORFs displayed similarity to characterized proteins. ORF44 (30259 to 31253) of the genome encoded an endolysin protein. Two tRNAs were found in the phage genome.

No virulence or lysogeny-related proteins were identified in the genome. Therefore, phage SM5 has the potential to be used as an alternative natural antibacterial agent to combat infections caused by multidrug-resistant (MDR) S. Typhi in clinical settings and for bioremediation of typhoid bacteria in sewage-contaminated water supplies in countries of endemicity like India.

### Data availability.

The genomic sequence of Salmonella phage vB_SenA_SM5 was deposited at NCBI GenBank under accession no. OM681507. The raw sequence data reads have been deposited at NCBI under SRA accession no. SRR18298590, BioProject accession no. PRJNA813368 and BioSample accession no. SAMN26288005.
